# Evaluation of PET Imaging Performance of the TSPO Radioligand [^18^F]DPA-714 in Mouse and Rat Models of Cancer and Inflammation

**DOI:** 10.1007/s11307-015-0877-x

**Published:** 2015-07-21

**Authors:** Jinzi Zheng, Alexandra Winkeler, Marie-Anne Peyronneau, Frédéric Dollé, Raphaël Boisgard

**Affiliations:** Inserm U1023, Laboratoire d’Imagerie Moléculaire Expérimentale, Université Paris Sud, Orsay, France; CEA, DSV/I2BM, Service Hospitalier Frédéric Joliot, Orsay, France; Techna Institute, University Health Network, 101 College Street, Room 7-302, Toronto, Ontario Canada M5G 1L7; Institute of Biomaterials and Biomedical Engineering, University of Toronto, Toronto, Ontario Canada

**Keywords:** Cancer, Inflammation, PET imaging, TSPO, DPA-714

## Abstract

**Purpose:**

Many radioligands have been explored for imaging the 18-kDa translocator protein (TSPO), a diagnostic and therapeutic target for inflammation and cancer. Here, we investigated the TSPO radioligand [^18^F]DPA-714 for positron emission tomography (PET) imaging of cancer and inflammation.

**Procedures:**

[^18^F]DPA-714 PET imaging was performed in 8 mouse and rat models of breast and brain cancer and 4 mouse and rat models of muscular and bowel inflammation.

**Results:**

[^18^F]DPA-714 showed different uptake levels in healthy organs and malignant tissues of mice and rats. Although high and displaceable [^18^F]DPA-714 binding is observed *ex vivo*, TSPO-positive PET imaging of peripheral lesions of cancer and inflammation in mice did not show significant lesion-to-background signal ratios. Slower [^18^F]DPA-714 metabolism and muscle clearance in mice compared to rats may explain the elevated background signal in peripheral organs in this species.

**Conclusion:**

Although TSPO is an evolutionary conserved protein, inter- and intra-species differences call for further exploration of the pharmacological parameters of TSPO radioligands.

**Electronic supplementary material:**

The online version of this article (doi:10.1007/s11307-015-0877-x) contains supplementary material, which is available to authorized users.

## Introduction

The 18-kDa translocator protein (TSPO) is a member of the evolutionary conserved transporter protein family [1]. In animals, TSPO is primarily located on the outer mitochondrial membrane of organs involved in steroid synthesis such as the adrenal glands, testis and ovaries, and the pituitary glands [2]. In the central nervous system (CNS), TSPO levels are dramatically enhanced in immune cells such as microglia and reactive astrocytes during neuroinflammation [3–7]. High levels of TSPO expression have also been reported in macrophages and monocytes in the periphery [5, 8, 9], as well as in breast, colorectal, prostate, ovarian, brain, and liver cancer cell lines [10]. The pattern of TSPO expression suggests that this protein plays a role in the modulation of immunity, inflammation, and cell proliferation. TSPO is thus a suitable biomarker candidate for a number of inflammatory diseases and cancer [11], and it has also been explored as a prognostic marker [12, 13] and a therapeutic target [7, 14–16]. As a result, TSPO has generated much interest as an imaging target, and a large number of TSPO radioligands have been synthesized for positron emission tomography (PET) imaging including [^11^C]PK11195, [^11^C]DAA1106, [^11^C]PBR28, [^18^F]PBR06, [^11^C]SSR180575, and [^18^F]DPA-714 [3, 17, 18].

Successful preclinical imaging evaluations have triggered the clinical use of several TSPO radioligands in small cohorts of patients [19]. The majority of these preclinical studies investigated TSPO PET radiotracers in models of neuroinflammation (*e.g*., Alzheimer’s disease [20], stroke [21], multiple sclerosis [22], and glioma [23, 24]), generally in the rat due to the small size of the mouse brain. However, recent reports on the imaging of peripheral TSPO expression in pulmonary, liver, muscle, and bowel inflammation [25–29] and in breast cancer xenografts [30, 31] have increased the interest in using TSPO ligands for imaging peripheral inflammation and cancer. The emergence of TSPO as a popular target for molecular imaging of major pathologies fosters the testing and validation of TSPO PET radioligands in an increasing number of animal models. However, the recent revelation that, in humans, a significant proportion of individuals (~14 %) who show *in vitro* specific binding for [^11^C]PK11195 do not bind [^11^C]PBR28 [32], and the lack of a clear causal relationship between *in vitro* affinity and *in vivo* binding of different TSPO radioligands in other species [33] raises legitimate concerns on the relevance of preclinical TSPO imaging for translational purposes.

The TSPO radioligand [^18^F]DPA-714 has been shown to possess excellent specificity and imaging performance in rat models of acute neuroinflammation, and initial evaluation in seven healthy human volunteers confirmed its biodistribution profile [34, 35]. [^18^F]DPA-714 is also the radioligand for PET imaging of TSPO that was selected for multicenter studies by the European consortium INMiND (imaging of neuroinflammation in neurodegenerative diseases) [36]. More recently, Lavisse *et al*. [37] reported that its binding in healthy human volunteers is influenced by the subject’s TSPO genotype (*i.e*., high, low, and mixed-affinity binders). In the present study, we investigate the biodistribution of [^18^F]DPA-714 and its ability to highlight TSPO expression in different mouse and rat models of cancer and peripheral inflammation.

## Materials and Methods

### Radiotracer Synthesis

*N*,*N*-Diethyl-2-(2-(4-(2-fluoroethoxy)phenyl)-5,7-dimethylpyrazolo[1,5-a]pyrimidin-3-yl)acetamide (DPA-714) was labeled with fluorine-18 at its 2-fluoroethyl terminal using a tosyloxy-for-fluorine nucleophilic aliphatic substitution slightly modified from a previously published procedure [38–40]. Typically, from a 37 GBq [^18^F]fluoride batch, 5.6–7.4 GBq of [^18^F]DPA-714 with >95 % chemical and radiochemical purity was routinely obtained in 85 to 90 min with specific radioactivities (SRA) ranging from 62 to 244 GBq/μmol. Final formulation was adjusted in order to provide [^18^F]DPA-714 batches with high radioactive concentrations, allowing thus the use of the radiotracer in multiple and consecutive imaging sessions scheduled on a same day.

### Animal Models

All experiments were conducted in accordance with the European Union regulations on animal research. MDA-MB-231 and MCF-7 human breast cancer cell lines were purchased from ATCC-LGC Standards. Suspended in a 1:1 volume ratio of DMEM and Matrigel^TM^ (BD Biosciences, Le Pont de Claix, France), 5 × 10^6^ cells were subcutaneously injected into the flanks of female nude NMRI mice (Elevage Janvier, Le Genest Saint Isle Saint Berthevin, France). The mice inoculated with MCF-7 cells were implanted with a 17β-estradiol pellet (0.72 mg/pellet, 60-day release, Innovative Research of America, Sarasota, FL, USA) under their dorsal skin 24 h before tumor inoculation. The primary HBCx-12B xenografts were established on female nude NMRI mice by surgically implanting a small piece of a freshly resected HBCx-12B tumor obtained from a donor mouse bearing a patient-derived tumor (Institut Curie, Paris, France) [41]. The PyMT tumor model was established in female FVB/NRj mice (Elevage Janvier, Le Genest Saint Isle Saint Berthevin, France) by subcutaneously implanting a small piece of a freshly resected MMTV-PyMT tumor from the donor animal’s mammary fat pad. Intracranial tumor development consisting in a stereotactic slow infusion (over 5 min) of 1 μl of cell solution was performed on female Wistar rats and female NMRI nude mice with the administration of 2 × 10^5^ 9L rat glioma cells (stereotactic coordinates for nude mice were 2.5 mm lateral to bregma and 3 mm from the dural surface). In addition, 2 × 10^6^ 9L cells were injected subcutaneously into a different group of Wistar rats to induce peripheral 9L tumors. For the peripheral inflammation model, 1 μl turpentine oil was injected intramuscularly into the right inner thigh of female nude NMRI mice and 10 μl into Wistar rats. Imaging of these acute inflammatory lesions was conducted 24 h postinduction. The inflammatory bowel disease model of colitis in C57BL/6 mice and Wistar rats was established by introducing 2.5 % of dextran sulfate sodium (DSS) daily in the animals’ drinking water for 7–8 days.

### PET Imaging and Analysis

All PET acquisitions were conducted on a Focus 220 preclinical PET system (Siemens, Knoxville, TN, USA). [^18^F]DPA-714 was i.v. injected at a dose of 0.22 ± 0.04 MBq/g of body weight (bw) and 0.06 ± 0.03 MBq/g bw into the tail vein of 56 mice (mean body weight 32.3 ± 3.9 g; among which 16 mice bore bilateral HBCx-12B s.c. tumors and 5 mice bore bilateral MDA-MB-231 s.c. tumors) and 19 rats (mean body weight 376.8 ± 58.4 g; among which 4 rats bore bilateral s.c. 9L tumors), respectively. After decay correcting for the time gap between [^18^F]DPA-714 delivery, injection and imaging for each animal, as well as taking into account the batch-to-batch variation in specific radioactivity, the mean pmol of DPA-714 per g of body weight was calculated to be 27.0 ± 20.3 for mice (range 3.9–83.2 pmol/g) and 7.7 ± 11.4 for rats (range 0.7–36.4 pmol/g). Immediately following radiotracer administration, the animals were placed in a warm chamber set to 30 °C. At 30 min postinjection, the animals were anesthetized using a 2–3 % isoflurane/oxygen mixture and positioned on the imaging bed for a 30-min static PET acquisition (2 frames, 15 min each). Sinograms generated from these emission scans were normalized and corrected for scatter, attenuation, and radioactivity decay. Fourier rebinning (FORE) and an iterative 2D ordered subset expectation maximization (OSEM) algorithm (16 subsets, 4 iterations) was applied for image reconstruction. Data visualization, contouring, and analysis were performed using the Anatomist BrainVISA software package (http://www.brainvisa.info) using the 2nd frame of the PET acquisition (45–60 min post tracer injection). Regions of interests (ROIs) were drawn manually on the organs and tissues of interest. Specifically for muscle, the ROIs were placed on either the upper thigh or arm. All data is expressed as the mean ± standard deviation. Pharmacokinetics analysis was performed by fitting a bi-exponential (two-compartment) model to the blood time-activity curves (TACs) for each animal and calculating the distribution half-life (*t*_1/2*α*_) and elimination half-life (*t*_1/2*β*_) of [^18^F]DPA-714 (assuming no metabolism). [^18^F]DPA-714 uptake was expressed as %ID/g, lesion-to-muscle ratios of %ID/g, as well as using the standardized uptake value (SUV) calculated as (activity/g of tissue)/[(injected activity)/(g of body weight)].

### Metabolite Analysis and Measurement of [^18^F]DPA-714 in Plasma and Muscle

Plasma and muscle samples were obtained from 4 female FVB mice (21 ± 1 g each) and 4 male Wistar rats (306 ± 69 g) which had received an i.v. injection of 0.74 ± 0.07 MBq/g bw and 0.14 ± 0.03 MBq/g bw of [^18^F]DPA-714, respectively, 60 min before kill. In order to detect the intact form of [^18^F]DPA-714 in plasma and muscle at 60 min p.i. in both species, the injected dose per body weight was 5.4-fold higher in mice. The radioactivity in the plasma and muscle samples was measured using a cross-calibrated gamma counter (Cobra Quantum D5003, Perkin Elmer, Waltham, MA, USA). [^18^F]DPA-714 was quantified using a previously published tissue extraction and HPLC analysis plus counting method [42]. The radioactivity associated with [^18^F]DPA-714 was expressed as a fraction of the summed radioactivity associated with all radioactive peaks recorded on the radiochromatograms. All data was expressed as %ID/g, SUV, and mouse-to-rat ratios of SUV.

### Statistical Analysis

All statistical comparisons were performed using SPSS Statistics 22 software (IBM, Armonk, NY, USA) and the independent samples unequal variance *t* test.

## Results

### [^18^F]DPA-714 Uptake in Neoplastic and Inflammatory Lesions

**Fig. 1:**
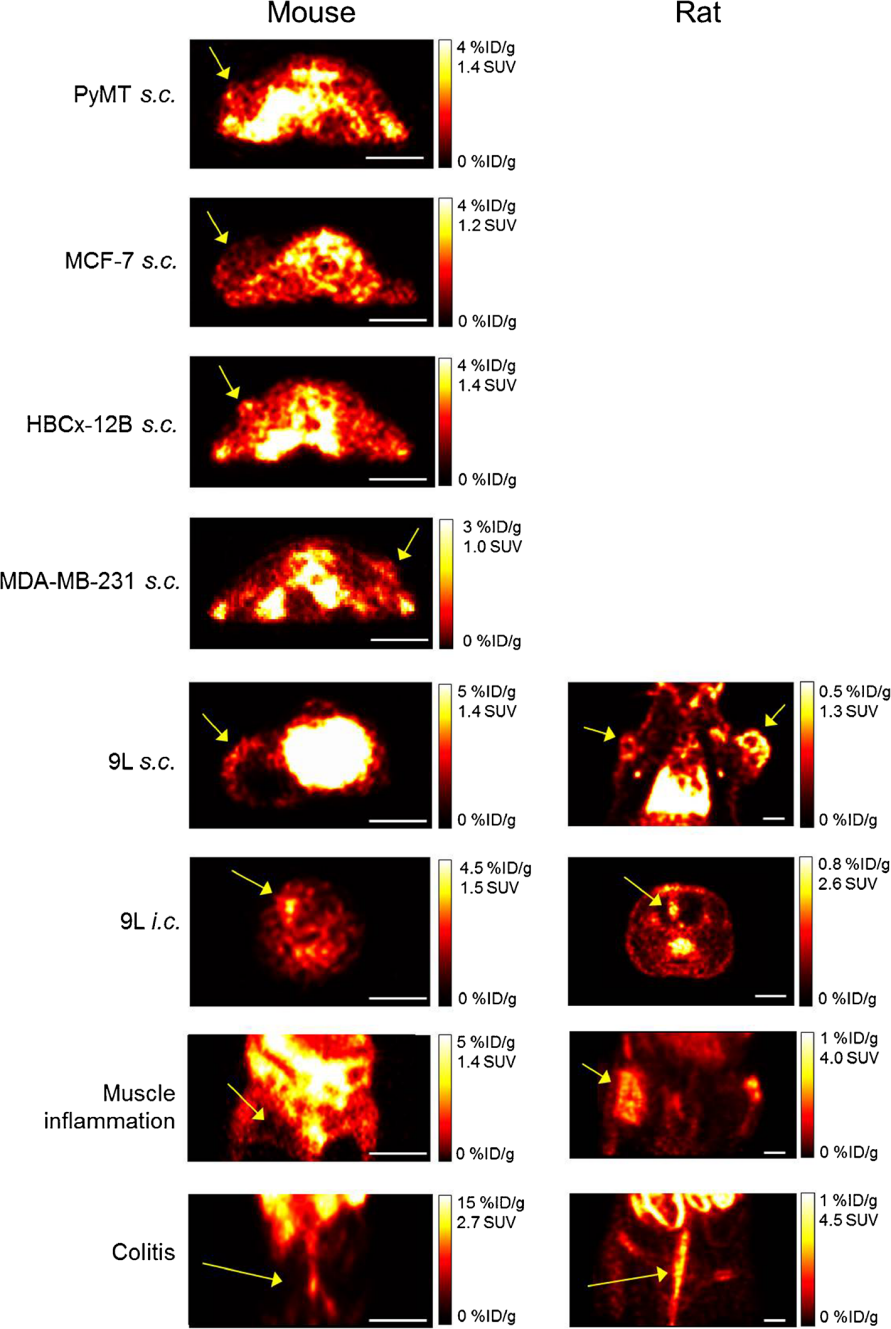
Representative PET images illustrating the uptake of [^18^F]DPA-714 at steady state (45 to 60 min postinjection) in different mouse and rat models of cancer and inflammation. *Yellow arrows* point to the lesions, and the *white bar* represents 1 cm.

**Fig. 2:**
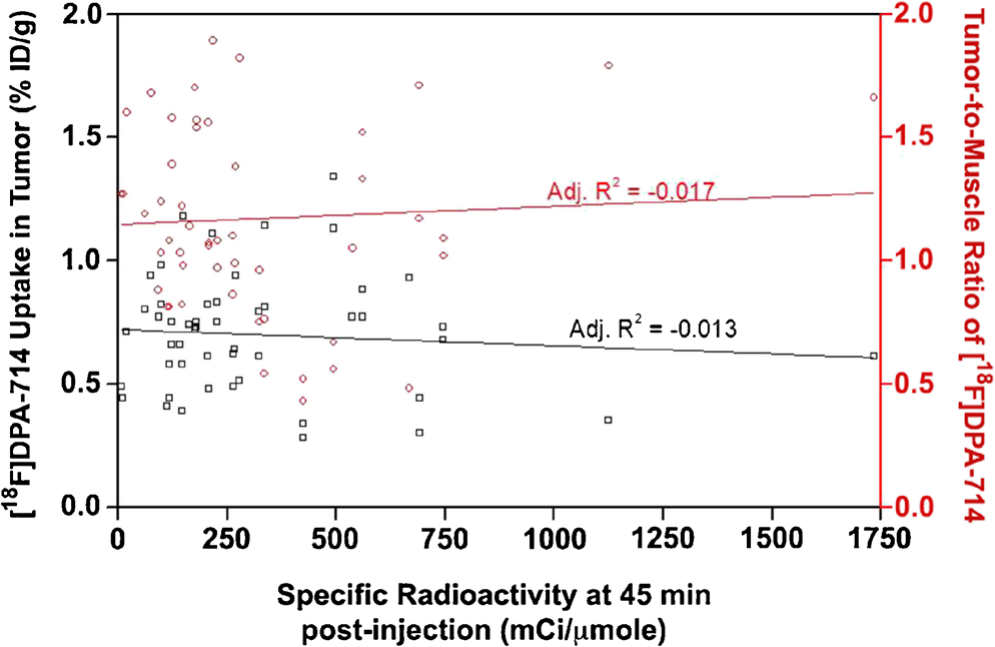
No correlation was found between the degree of [^18^F]DPA-714 uptake in subcutaneous mouse tumors or the tumor-to-muscle ratio and the specific radioactivity of [^18^F]DPA-714 at 45 min postinjection. The data presented was obtained from PET images from 36 independent imaging sessions (7 independent radiotracer productions) and 50 tumors.

The ability of [^18^F]DPA-714 to detect, visualize, and accumulate in cancer and inflammation lesions was evaluated in C57BL/6 and NMRI nude mice, as well as Wistar rats in a total of 8 different tumor models and 4 inflammatory models (Table 1). The uptake of [^18^F]DPA-714 in peripheral tumors was higher in mice than in rats (0.85 ± 0.36 %ID/g of tissue, *n* = 61, *versus* 0.23 ± 0.04 %ID/g of tissue, *n* = 8). However, due to the low peripheral tissue background signal, the lesions were much more easily identifiable in rats (Fig. 1) and the tumor-to-muscle ratios were significantly higher in rats than in mice (13.9 ± 1.8 compared to 1.2 ± 0.2). When normalized by the animals’ body weight, the mean SUV of subcutaneous tumors in mice was 0.27 ± 0.09 and that of rat was 0.71 ± 0.22. These differences were independent of the SRA of [^18^F]DPA-714 at time of image acquisition (45 min postinjection) as shown in Fig. 2. Here, we investigated a subset of animals and tumors, for which the SRA values for [^18^F]DPA-714 from 7 separate productions used for the 36 PET imaging sessions ranged from 44 to 1732 mCi/μmol. The SRA values (decay corrected to 45 min p.i.) showed no correlation with tracer uptake values and tumor-to-muscle ratios in 50 different tumors (Fig. 2). Similarly, the uptake of [^18^F]DPA-714 in the two peripheral inflammatory models investigated were higher in mice than in rats. However, the lesion-to-control ratios were significantly higher in rats (9.1 ± 0.8) than in mice (1.1 ± 0.6). Specifically, the control regions employed were the healthy muscle contralateral to the turpentine oil-induced inflammation site for the muscle inflammation model and the distal colon in healthy animals for the inflammatory bowel disease model. It is noteworthy that previous results showed significant and displaceable [^18^F]DPA-714 binding *ex vivo* in all four subcutaneously implanted mouse tumor models investigated here (PyMT, MCF-7, HBCx-12B, and MDA-MB-231) [43]. In contrast, intracranially implanted tumors were easily detectable in both mice and rats. Using a mirror ROI contralateral to the tumor area as control, the cerebral tumor lesion-to-control ratios were similar between the two species (2.3 ± 0.3 in mice and 3.1 ± 0.9 in rats), confirming the feasibility of utilizing [^18^F]DPA-714 for imaging of CNS lesions in both mice and rats.

**Table 1:** *In vivo* uptake of [^18^F]DPA-714, expressed as %ID/g of tissue and target-to-control ratios as well as in SUV, in lesions of cancer and inflammation in various mouse and rat disease models

Animal model	Lesion TSPO expression level and *ex vivo* [^18^F]DPA-714 binding	Number of animals	*In vivo* PET lesion [^18^F]DPA-714 uptake (% ID/g)	*In vivo* PET lesion-to-control ratio of [^18^F]DPA-714	*In vivo* mouse-to-rat [^18^F]DPA-714 uptake ratio	*In vivo* mouse-to-rat lesion-to-control ratio	*In vivo* PET SUV
PyMT s.c. tumor (nude mouse)	++ [43]	5	0.6 ± 0.2	1.5 ± 0.3			0.22 ± 0.06
MCF-7 s.c. tumor (nude mouse)	+++ [43]	7	0.8 ± 0.3	1.0 ± 0.5			0.24 ± 0.08
HBCx-12B s.c. tumor (nude mouse)	++ [43]	32	0.7 ± 0.2	1.2 ± 0.4			0.24 ± 0.09
MDA-MB-231 s.c. tumor (nude mouse)	++ [43]	10	0.6 ± 0.2	1.1 ± 0.4			0.22 ± 0.07
9L s.c. tumor (nude mouse)	++ [23]	7	1.5 ± 0.3	1.3 ± 0.4	6.5	0.10	0.43 ± 0.08
*9L s.c. tumor (Wistar rat)*		*8*	*0.2 ± 0.04*	*13.9 ± 1.8*			*0.71 ± 0.22*
9L i.c. tumor (nude mouse)		4	2.0 ± 0.4	***2.3 ± 0.3***	5.8	0.74	0.71 ± 0.15
*9L i.c. tumor (Wistar rat)*		*8*	*0.4 ± 0.1*	*3.1 ± 0.9*			*1.07 ± 0.25*
Turpentine oil i.m. inflammation (nude mouse)	+ [29]	6	0.5 ± 0.2	0.6 ± 0.2	1.2	0.07	0.13 ± 0.05
*Turpentine oil i.m. inflammation (Wistar rat)*	*+* *(Fig. S* *1* *)*	*4*	*0.4 ± 0.04*	*8.5 ± 3.6*			*1.56 ± 0.16*
Inflammatory bowel disease (C57BL/6 mouse)	+	6	3.9 ± 2.0	1.5 ± 0.7	13.0	0.16	0.71 ± 0.45
*Inflammatory bowel disease (Wistar rat)*	*+ [* 28 *]*	*3*	*0.3 ± 0.1*	*9.6 ± 4.8*			*1.50 ± 0.14*
***Mean ± std across all lesions (mouse)***		77	1.3 ± 1.2	1.3 ± 0.5			
***Mean ± std across all lesions (rat)***		23	0.3 ± 0.1	***8.8 ± 4.5***			

The lesion TSPO expression levels were quantified using Western blot (sum of human and murine TSPO). The *ex vivo* [^18^F]DPA-714 binding was assessed using autoradiography of tissue sections and all lesions demonstrated specific and displaceable binding in the presence of an excess of unlabeled DPA-714 or PK11195. Rat models are italized and lesions with high target-to-control ratios (≥2.0) are in bolditalic. Note: The control region used for the intracranial (i.c.) tumors is an ROI of equal volume in the contra-lateral brain hemisphere. The control region used for the inflammatory bowel disease model is the distal colon of healthy animals. The control region used for all other lesions is muscle. All lesions were tested for *ex vivo* [^18^F]DPA-714 binding and showed specific binding displaceable by unlabeled DPA-714 and PK11195

*s.c*. subcutaneous, *i.c*. intracranial, *i.m*. intramuscular

### Biodistribution of [^18^F]DPA-714 in Mice and Rats

**Fig. 3:**
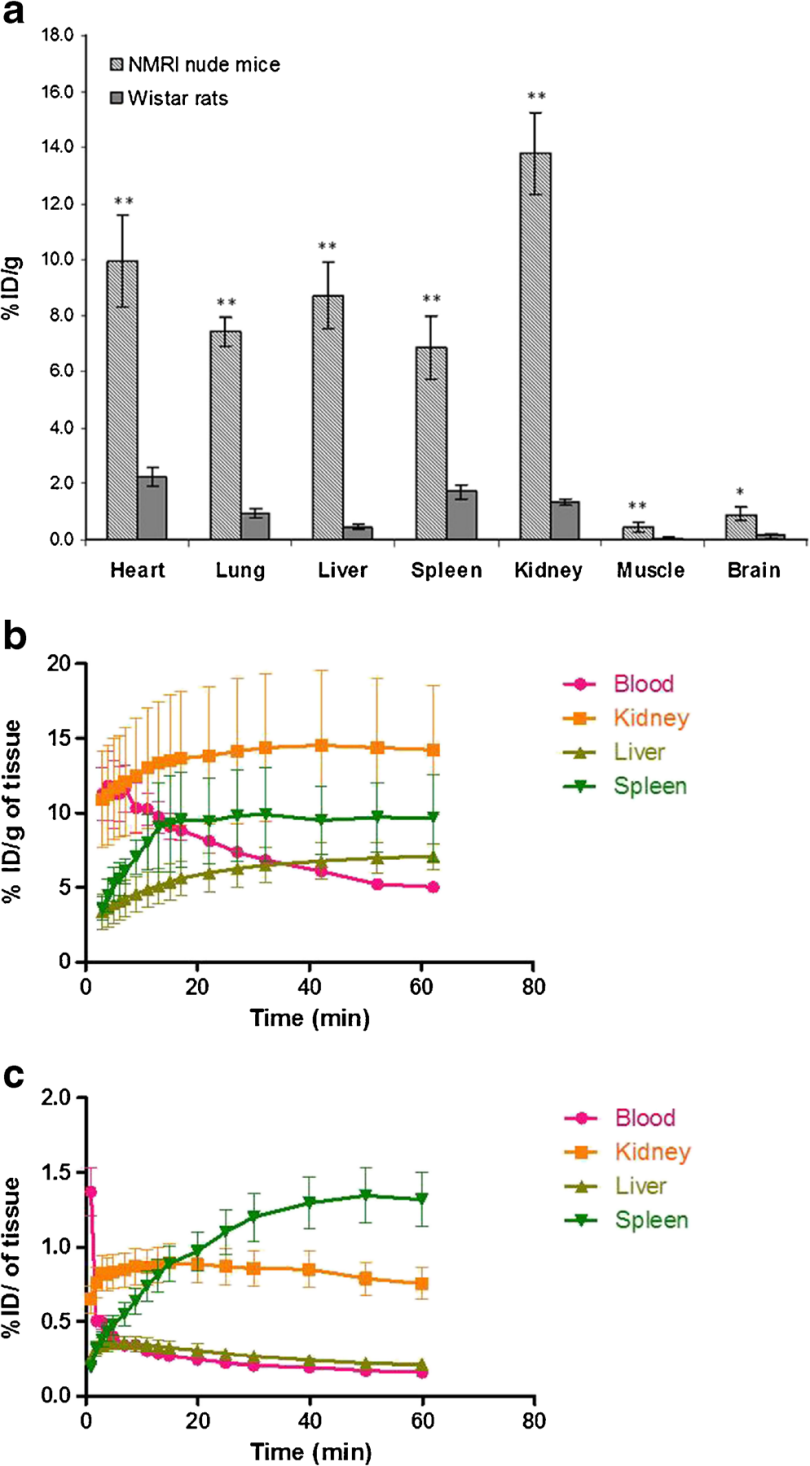
**a** Distribution and uptake of [^18^F]DPA-714 (%ID/g of tissue) in organs of healthy mice and rats at 45 to 60 min postinjection measured from the PET data sets. In all peripheral organs investigated, the difference in tracer distribution between mice and rats was highly statistically significant (denoted with **p < 0.001). In the brain, the *p* value calculated following an independent samples *t* test performed on the mice *vs*. rats values was 0.002 (denoted with *). The same data plotted as SUV can be found in Supplementary Figure S2. **b** Time-activity curves (TACs) obtained from the blood, kidney, liver, and spleen of mice (*n* = 3) and **c** rats (*n* = 2) showing a more prolonged vascular half-life of [^18^F]DPA-714 (and possibly its radiometabolites) in mice as well as a higher level of radioactivity detected in all organs included in this analysis over the first 60 min postinjection.

Figure 3a and Supplementary Fig. S2 show the uptake of [^18^F]DPA-714 at steady state (measured at 45 to 60 min postinjection using the PET data set) in organs and tissues of healthy NMRI nude mice and Wistar rats. Compared to rats, mice presented a statistically higher mean %ID/g of tissue in all organs and tissues investigated; the ratios of mean %ID/g of tissue of uptake between mice (*n* = 5) and rats (*n* = 4) were 4.4 (heart), 7.7 (lungs), 19.2 (liver), 4.0 (spleen), 10.1 (kidneys), 7.2 (muscle), and 6.2 (brain). The independent samples *t* test showed that the statistical difference in accumulation at steady state in mice compared to rats was higher in the peripheral organs (*p* < 0.001) compared to the brain (*p* = 0.002). This difference is not due to the higher dose of radioactivity (*i.e*., [^18^F]DPA-714) normalized to body weight administered to mice compared to rats (0.17 ± 0.06 MBq/g bw *versus* 0.03 ± 0.01 MBq/g bw), or the amount of DPA-714 administered to mice (22.3 ± 7.8 pmol/g bw) compared to rats (0.9 ± 0.1 pmol/g bw). In fact, in the muscle, an organ of low TSPO expression in both species, the TACs (Supplementary Fig. S3) acquired from 0 to 60–70 min following i.v. administration of [^18^F]DPA-714 showed no correlation between the uptake and the injected dose per body weight of total DPA-714 (radiolabeled and non-radiolabeled) in the range of 2.7 to 36.4 pmol/g. However, a clear difference in the muscle uptake levels of the tracer between the two species exists. Figure 3b and c shows the TACs for 4 additional healthy tissues and organs including the blood, kidney, liver, and spleen in the two species during the first 60 min of [^18^F]DPA-714 uptake. It can be observed that both the tracer distribution and elimination half-lives are much longer in mice (*t*_1/2*α*_ = 15.6 ± 2.8 min, *t*_1/2*β*_ = 242.2 ± 955.9 min, *R*^2^ = 0.97 ± 0.02) compared to rats (*t*_1/2*α*_ = 0.3 ± 0.1 min, *t*_1/2*β*_ = 31.2 ± 3.6 min, *R*^2^ = 0.98 ± 0.00).

### Metabolism of [^18^F]DPA-714 in Mice and Rats

**Fig. 4:**
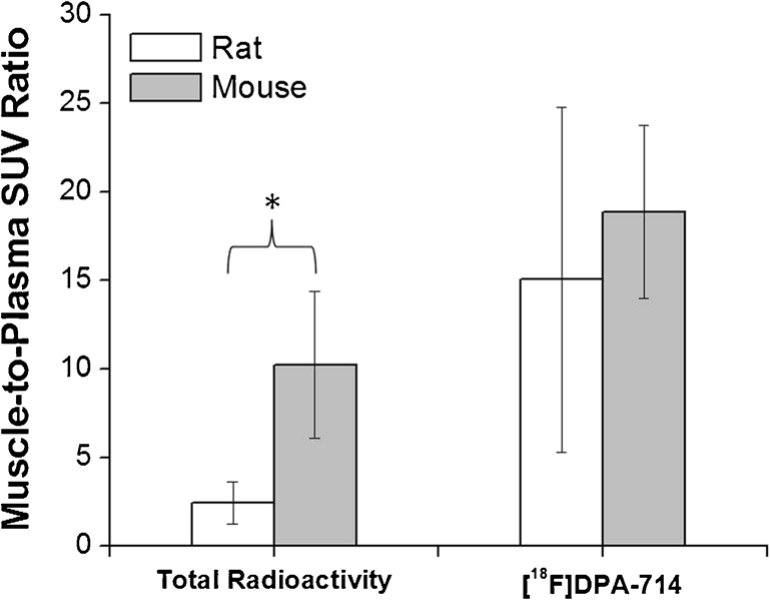
Muscle-to-plasma SUV ratios of total radioactivity and [^18^F]DPA-714 measured at 60 min postinjection in mice and rats using HPLC-based radiochromatography. The *data bars* represent the mean and standard deviation calculated from four animals. * denotes *p* < 0.05.

*Ex vivo* radio-HPLC analysis of the mouse and rat plasma and muscle performed at 60 min postinjection showed species-dependent differences in radiotracer clearance and metabolism. Specifically, the proportions of intact [^18^F]DPA-714 detected in the mouse and rat muscle were 97.3 ± 1.7 % and 85.3 ± 6.2 % of the total radioactivity, respectively (*p* = 0.026). Similarly in plasma, the proportions of intact [^18^F]DPA-714 detected were 52.3 ± 18.4 % and 16.0 ± 5.1 % of total radioactivity for mice and rats, respectively (*p* = 0.025). These statistically significant differences illustrate the faster metabolism of [^18^F]DPA-714 in rats compared to mice. In addition, direct comparison of the total amount of radioactivity measured in the mouse and rat muscle shows that the overall mouse muscle radioactivity is higher than in rat (mean muscle SUV corresponding to 0.67 ± 0.73 and 0.43 ± 0.13, respectively), while the total radioactivity measured in plasma was higher in rat than in mouse (mean plasma SUV of 0.21 ± 0.14 *vs*. 0.07 ± 0.07). These observations illustrate that despite a faster clearance of overall radioactivity ([^18^F]DPA-714 plus its radiometabolites) from mouse plasma compared to rats, the muscle radioactivity build-up is significantly higher in mice. In fact, the muscle-to-plasma ratio for total radioactivity expressed as SUV was calculated to be 10.2 ± 4.2 in mice and 2.5 ± 1.2 in rats (*p* = 0.029, Fig. 4). As a result, the higher amount of total radioactivity and intact [^18^F]DPA-714 present at 60 min p.i. in the mouse muscle compared to rat muscle is believed to be a significant contributor to the low contrast observed between the peripheral tumor/inflammation areas and the surrounding healthy muscle tissue in the PET images acquired in mice.

## Discussion

The present results show that even though TSPO is evolutionary conserved and its expression is boosted in inflammation and cancer in both mice and rats, the *in vivo* imaging performance of [^18^F]DPA-714 is clearly species dependent. In mice, in two peripheral disease models of inflammation (*n* = 6) and five models of subcutaneously implanted tumors (*n* = 5 to 32), no significant target-to-background ratios of [^18^F]DPA-714 were measured in peripheral murine disease tissues *in vivo* using PET imaging despite a high TSPO expression relative to control regions had been measured using Western blot and *ex vivo* autoradiography [43]. However, the same disease type in the same tissue (*i.e*., subcutaneously implanted breast or glioma tumors, turpentine oil-induced peripheral inflammation and inflammatory bowel disease) provided a high uptake of [^18^F]DPA-714 only in rats (Table 1, Fig. 1). The higher overall radioactivity signal measured in the mouse muscle and the higher percentage of intact radioligand detected in mouse plasma and muscle compared to rat suggest that plasma metabolism and muscle clearance of [^18^F]DPA-714 are delayed in mice relative to rats. The longer vascular half-life profile of [^18^F]DPA-714 and its radiometabolites in mice compared to rats was also confirmed *in vivo* by TACs obtained in the first 60 min post tracer administration (Fig. 3b, c). Slow plasma metabolism and pharmacokinetics of [^18^F]DPA-714 may cause a build-up of radioactivity and result in a high non-specific background signal, as seen in the PET images in mice, decreasing lesion-to-background contrast. This hypothesis is supported by previously published results which demonstrated minimal level of [^18^F]DPA-714 binding *ex vivo* in mouse muscle tissue compared to high TSPO-expressing tumor tissues [43]. A possible explanation of why this build-up of free radioligand would spare the brain could be that [^18^F]DPA-714 and most of its radiometabolites do not easily cross the intact blood brain barrier. This explanation is consistent with the low background signal associated with [^18^F]DPA-714 in the healthy brain [35, 44]. Other possible factors that can contribute to the species differences observed in this study include differential binding affinity of [^18^F]DPA-714 for mouse and rat TSPO, as well as potential presence of [^18^F]DPA-714-binding sites in mice not related to TSPO. In addition, preclinical disease models often require the use of immune-compromised animals, such as nude mice, in this study as hosts to human-derived cancer cells. Physiological differences stemmed from differing immune systems between these immune-compromised animals and their wild-type counterparts may also be a contributor to the observed differential radiotracer performance. As a result, further investigations are necessary to fully understand and characterize the differences in clearance and metabolism of [^18^F]DPA-714 observed in mice and rats, as is also important to determine whether other currently available TSPO radioligands share the same species-dependent pharmacokinetics and metabolism characteristics as [^18^F]DPA-714.

## Conclusions

This report confirms the suitability of [^18^F]DPA-714 as an effective PET imaging probe for cancer and inflammation in both the CNS and periphery of rats and CNS lesions in mice. However, significant differences in species-dependent metabolism and muscle accumulation of [^18^F]DPA-714 caution the use of this radiotracer, and possibly other TSPO-targeted probes, for imaging TSPO-positive peripheral lesions in mice.

## Electronic supplementary material

ESM 1 (PDF 2696 kb)
