# Supplementary material for: Evaluation of PET Imaging Performance of the TSPO Radioligand [18F]DPA-714 in Mouse and Rat Models of Cancer and Inflammation
Source: Mol Imaging Biol. 2015 Jul 21;18:127–34. doi: 10.1007/s11307-015-0877-x (PMC4722075; doi:10.1007/s11307-015-0877-x)

Evaluation of PET Imaging Performance of the TSPO Radioligand [^{18}F]DPA-714 in Mouse and Rat Models of Cancer and Inflammation

Jinzi Zheng [1,2,3,4*], Alexandra Winkeler [1,2], Marie-Anne Peyronneau[2], Frédéric Dollé[2], Raphaël Boisgard[1,2*]

Journal: Molecular Imaging and Biology

Author affiliations :

1. Inserm U1023, Laboratoire d'Imagerie Moléculaire Expérimentale, Université Paris Sud, Orsay, France
2. CEA, DSV/I^{2}BM, Service Hospitalier Frédéric Joliot, Orsay, France
3. Techna Institute, University Health Network, Toronto, Ontario, Canada
4. Institute of Biomaterials & Biomedical Engineering, University of Toronto, Ontario, Canada

*Corresponding authors:

Dr. Jinzi Zheng, Techna Institute, University Health Network, 101 College Street, Room 7-302, Toronto, Ontario, Canada M5G 1L7. Phone: +1 416 581 7790. Fax: +1 416 260 9709. E-mail: jinzi.zheng@rmp.uhn.on.ca.

Dr. Raphaël Boisgard, Inserm U1023, CEA-DSV-I^{2}BM, Service Hospitalier Frédéric Joliot, 4 Place du Général Leclerc, 91400 Orsay Cedex, France. Phone: + 33 1 69 86 78 01. Fax: +33 1 69 86 77 86. E-mail: raphael.boisgard@cea.fr.

Supplementary Data

Figure S1. (a) Western blot of rat adrenal gland (positive control), healthy muscle (negative control) and inflamed muscle tissue at 24 hours post turpentine oil injection, and (b) immunohistochemistry of rat inflamed muscle tissue at 72 hours post turpentine oil injection.

(a)

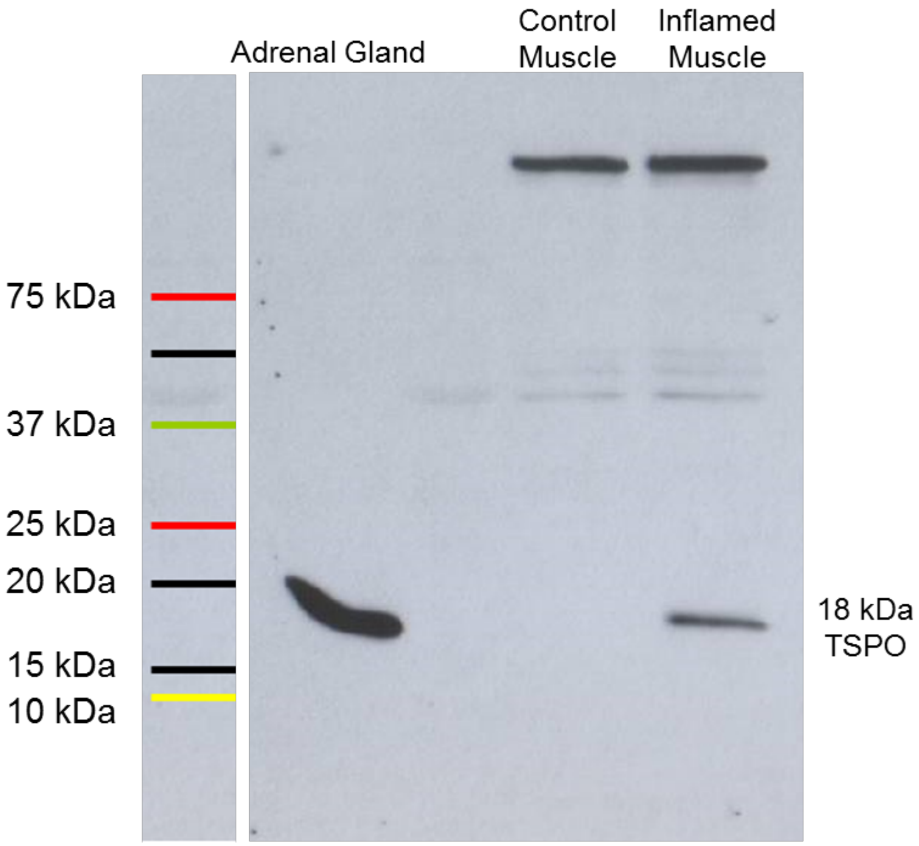

(b)

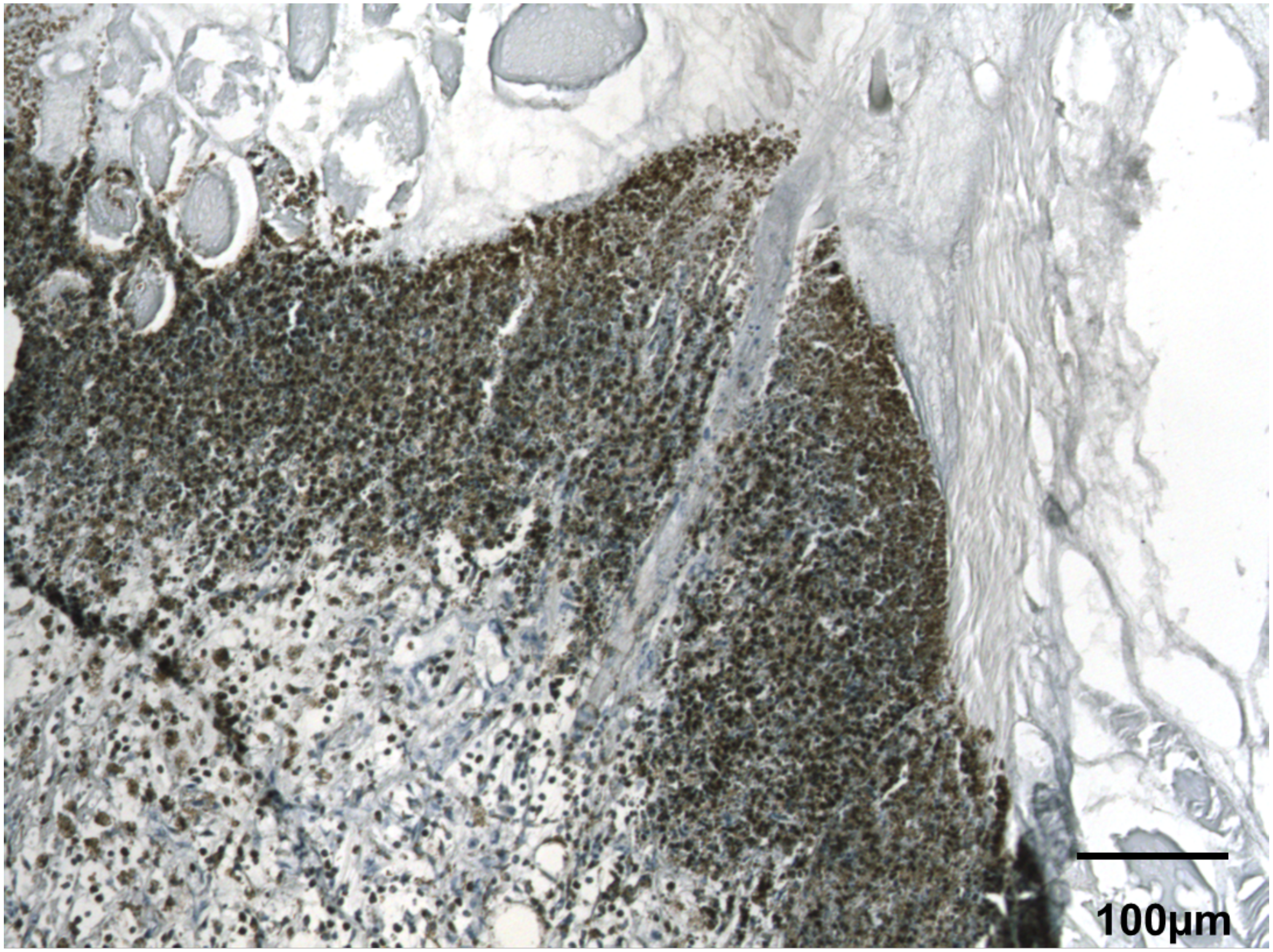

Figure S2. Distribution and uptake of [^{18}F]DPA-714 (SUV) in organs of healthy mice and rats at 45 to 60 minutes post-injection measured from the PET data sets.

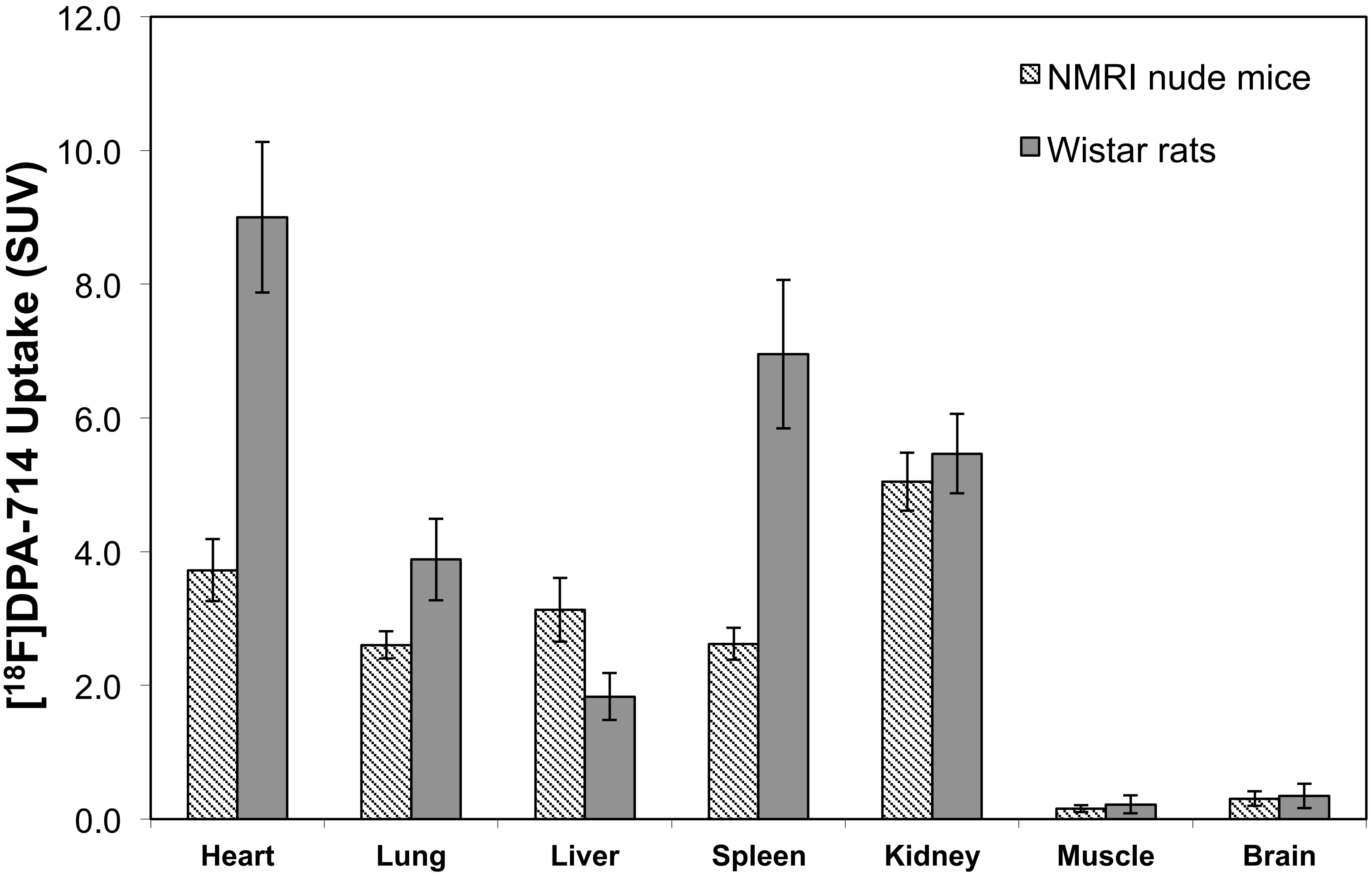

Figure S3. Distribution kinetics of [^{18}F]DPA-714 in healthy muscle of mice and rats (0 – 60/70 minutes post-injection). The [^{18}F]DPA-714 muscle uptake and uptake kinetics are independent of the injected dose of total DPA-714 (radiolabelled and non-radiolabelled) in the range of approximately 3-36 pmol/g bw for mice and rats. However, these two parameters are significantly different between the two species investigated. Specifically in mice, the muscle time-activity curve (TAC) shows a perfusion-induced peak within the first 10 minutes followed by a slow increase in PET signal due to potential non-specific tracer build-up.

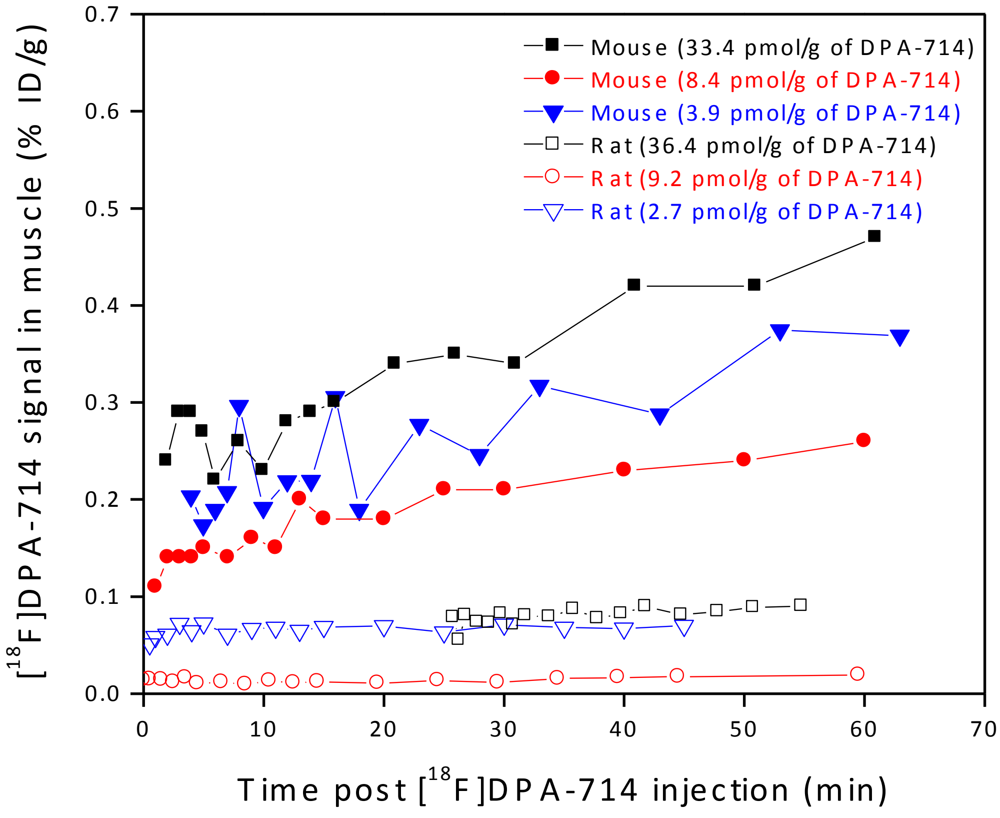

Supplement: Supplementary file 1 — (PDF 2696 kb) [file 11307_2015_877_MOESM1_ESM.pdf]
